# Adaptation and Validation of the Scale for Chinese Preschool Teachers’ Self-Efficacy (SCPTSE): Based on Classical Test Theory and Item Response Theory

**DOI:** 10.3390/bs15060741

**Published:** 2025-05-27

**Authors:** Hao Lu, Xiumin Li, Kejian Li

**Affiliations:** 1 College of Child Development and Education, Zhejiang Normal University, Hangzhou 311231, China; nelsonluh@163.com; 2 Faculty of Preschool and Special Education, Xuzhou Kindergarten Teachers College, Xuzhou 221004, China

**Keywords:** self-efficacy, Chinese in-service preschool teachers, cross-cultural adaptation, psychometric properties

## Abstract

Preschool teachers’ self-efficacy is essential to improve their professional development and the quality of early childhood education. This study adapted and validated the Scale for Chinese Preschool Teachers’ Self-Efficacy (SCPTSE) based on the Ohio State Teacher Efficacy Scale (OSTES), in accordance with Bandura’s Social Cognitive Theory. Following a rigorous four-stage cross-cultural adaptation procedure, the 21-item SCPTSE was administered to 882 in-service preschool teachers from Zhejiang, Henan, and Shaanxi provinces in China (M age = 30.41, SD = 6.05). Both CTT and IRT frameworks were employed to evaluate the scale’s psychometric properties. Under CTT, the SCPTSE demonstrated high internal consistency (α = 0.980), and CFA supported a robust three-factor structure—instructional strategies, classroom management, and child engagement—with excellent model fit (RMSEA = 0.079; SRMR = 0.025; CFI = 0.953; TLI = 0.947; NFI = 0.945; IFI = 0.953; PNFI = 0.837; PGFI = 0.700). Under IRT, all three sub-scales demonstrated strong unidimensionality (H_IS_ = 0.812, H_CM_ = 0.800, H_CE_ = 0.818), the SCPTSE’ items demonstrate excellent discrimination capabilities(all a > 1.70), overall reasonable difficulty(*b*_1_ < *b*_2_ < *b*_3_ < *b*_4_), and balanced information distribution. Nevertheless, the relatively low-difficulty design (e.g., *b*_1_) indicates room for improvement. Notably, cultural adaptation efforts ensured the scale’s contextual relevance to China’s preschool education system. The SCPTSE thus offers a valid, reliable, and culturally responsive tool for assessing self-efficacy of Chinese in-service preschool teachers and holds promise for informing targeted professional development and comparative international research.

## 1. Introduction

Self-efficacy, a central concept in social cognitive theory, refers to an individual’s belief in their ability to organize and execute actions necessary to achieve specific goals (Bandura, 1997). It influences behavior, goal-setting, and responses to challenges, and shapes cognitive patterns and emotional reactions, ultimately shaping personal development and environmental adaptation (Tschannen-Moran & Hoy, 2007; Bandura, 2009, 2011a; Lyons & Bandura, 2019). In education, teacher self-efficacy is defined as “teachers’ beliefs in their capabilities to organize and execute courses of action required to successfully accomplish specific teaching tasks in particular contexts” (Tschannen-Moran et al., 1998, p. 233). According to the self-fulfilling prophecy model, teachers with high self-efficacy set ambitious goals, invest greater effort, and continually refine their teaching practices, while those with lower self-efficacy are more susceptible to self-doubt and burnout (Tschannen-Moran & Hoy, 2007; Rimm-Kaufman & Hamre, 2010; Zee & Koomen, 2016). Thus, self-efficacy is crucial in making effective teaching both achievable and sustainable.

From a functional perspective, teacher self-efficacy impacts educational quality on micro and macro levels. At the classroom level, it directly influences instructional decisions and teaching practices (Tschannen-Moran & Hoy, 2007). On a macro level, it predicts students’ academic achievement and motivation, and it is closely associated with teachers’ professional commitment, retention, resilience, and mental health (Caprara et al., 2006; W. Y. Chan et al., 2008; Knobloch & Whittington, 2002; Mu et al., 2024). Zee and Koomen’s (2016) systematic review further confirms that higher self-efficacy correlates with improved classroom management and increased student engagement and achievement, and enhances occupational well-being. Thus, teacher self-efficacy is a vital personal resource that drives motivation and supports ongoing professional development.

Drawing on Bandura’s (1995) framework—comprising mastery experiences, vicarious experiences, verbal persuasion, and physiological and emotional regulation—teacher self-efficacy is a key internal force driving sustained professional and self-directed growth (Bandura, 1995; Lyons & Bandura, 2019). In teaching, especially early childhood education (ECE), self-efficacy supports development. Teachers with high self-efficacy tend to excel in refining their practice through self-reflection, a crucial skill in ECE settings. The growing importance of teacher self-efficacy in ECE has garnered widespread recognition (e.g., Guo et al., 2010, 2011; Wolstein et al., 2021; Yin et al., 2022). Preschool teachers, who must address not only the individual differences in children’s development but also the complexities of home–school cooperation and administrative burdens (Kluczniok et al., 2013; Hall-Kenyon et al., 2014), face multifaceted challenges. Consequently, deepening our understanding of preschool teacher self-efficacy is essential to improve their professional development and the quality of early childhood education.

### 1.1. Teacher Self-Efficacy: Theory and Measurement

The development of teacher self-efficacy measurement tools stems primarily from two theoretical frameworks: Rotter’s (1966) social learning theory and Bandura’s (1977) social cognitive theory. Based on Rotter’s social learning theory, researchers developed instruments centered around the concept of locus of control, attributing academic outcomes to internal or external factors. For instance, Armor et al. (1976) proposed the Rand Measure, which assesses teachers’ beliefs regarding the influence of the home environment (general teaching efficacy, GTE) and personal ability (personal teaching efficacy, PTE) using only two items. Although the simplicity of this tool laid the groundwork for subsequent research, its reliability has been widely debated. Guskey’s (1981) Responsibility for Student Achievement (RSA) questionnaire required teachers to allocate responsibility weights for student success (R^+^) and failure (R^−^), but its complexity limited its use. Similarly, Rose and Medway’s (1981) Teacher Locus of Control Scale (TLC) introduced context-specific items to explore causal attributions for teaching outcomes but suffered from an unstable factor structure. Overall, these tools based on Rotter’s theory tend to oversimplify teacher efficacy as a binary internal–external attribution, failing to capture its multidimensional nature.

Bandura’s social cognitive theory revolutionized efficacy measurement by distinguishing self-efficacy from outcome expectations and emphasizing its situational specificity. Building on this, Gibson and Dembo’s (1984) Teacher Efficacy Scale (TES) introduced a 30-item tool divided into PTE and GTE, though it faced criticism for cross-loadings and conceptual ambiguity (Tschannen-Moran et al., 1998). Rubeck and Enochs (1991) later developed the Science Teaching Efficacy Belief Instrument (STEBI), which, by designing subject-specific items, validated the multidimensional structure of efficacy and became a standard tool in science education. Meanwhile, Bandura’s unpublished Teacher Self-Efficacy Scale (TSES) broadened the measurement dimensions by incorporating seven areas—including classroom management and home–school collaboration—but remains underutilized due to limited evidence of reliability and validity.

To bridge the gap between theory and measurement, Tschannen-Moran et al. (1998) and Tschannen-Moran and Hoy (2001) expanded the concept of TSE and developed an integrated model that blends Bandura’s concept of self-efficacy and outcome expectations. This model offers a more comprehensive view of TSE by addressing the practical challenges teachers face across their professional roles. Based on this framework, Tschannen-Moran and Hoy (2001) developed the Ohio State Teacher Efficacy Scale (OSTES), which reflects Bandura’s framework (Klassen & Chiu, 2011) and has undergone rigorous validation through empirical research. OSTES is available in two forms—24-item and 12-item versions—and assesses three core dimensions: efficacy for instructional strategies, efficacy for classroom management, and efficacy for student engagement. It demonstrates strong internal consistency and significant positive correlations with existing measurement tools, while also demonstrating discriminant validity through its negative associations with job alienation and an external locus of control (Tschannen-Moran & Hoy, 2001). By balancing situational specificity with conceptual breadth, OSTES captures the core instructional responsibilities of teachers without being overly narrow in scope, thereby avoiding the limitations of prior instruments. As such, OSTES has become a widely adopted and empirically robust tool in contemporary research on teacher self-efficacy (Ruan et al., 2015).

### 1.2. Cross-Cultural Studies of Ohio State Teacher Efficacy Scale (OSTES)

Originally designed for American teachers, the OSTES has gained widespread recognition in educational psychology for its theoretical rigor and cross-cultural applicability. Klassen et al. (2009) employed multi-group confirmatory factor analysis (CFA) with samples from Canada, Cyprus, the United States, South Korea, and Singapore, confirming the reliability and measurement invariance of the TSES and providing a solid empirical basis for comparing the construct of teacher efficacy across diverse cultural contexts. Similarly, Tsigilis et al. (2010) validated the Greek version of TSES, demonstrating sound factor structure, internal consistency, temporal stability, and correlation with external standards using both within- and between-construct methods. Meanwhile, Nie et al. (2012) revised and further tested the TSES, confirming its predictive and construct validity in a sample of Singaporean teachers, which supported the scale’s applicability in Southeast Asia. Additionally, Burgueño et al. (2018) compared three versions of TSES—the TSES-24, TSES-12, and TSES-11. Findings showed that the 11-item version outperformed others in tests of gender invariance. Its overall factor and sub-factors positively predicted intrinsic motivation and life satisfaction, and negatively correlated with amotivation, thereby validating the applicability of the TSES-11 three-factor model for pre-service teachers.

Dominguez-Lara et al. (2019) used confirmatory factor analysis and exploratory structural equation modeling (ESEM) to examine the TSES in Peruvian public school teachers. They found that the traditional three-factor model did not achieve an ideal fit in this sample, whereas a two-factor model demonstrated higher reliability and validity for both male and female groups. This finding suggests that cultural and educational contexts may significantly influence the structure of teacher self-efficacy constructs. W. T. Chan et al. (2024) found that early childhood teachers’ efficacy beliefs were best captured by a bifactor model, comprising one general factor and three specific factors. The results suggest that teachers tend to conceptualize their teaching efficacy more broadly, emphasizing general teaching competence over domain-specific classroom functions.

In East Asian contexts, Ruan et al. (2015) validated both the long and short forms of TSES using samples from China, South Korea, and Japan. Their research revealed that the 24-item version did not fit the three-factor model well across countries, while the 12-item version demonstrated acceptable fit indices. Based on these findings, the researchers further proposed and validated an 11-item three-factor model. Measurement invariance tests confirmed its suitability for comparing teacher efficacy beliefs among China, South Korea, and Japan, thus providing an effective tool for cross-cultural comparative research.

In mainland China, Shao et al. (2025) used the OSTES to assess pre-service preschool teachers’ self-efficacy, confirming its strong structural validity, internal consistency, and measurement invariance. In Hong Kong, H. Cheung (2006) and Tsui and Kennedy (2009) independently employed the Chinese version of the 12-item Teacher Self-Efficacy Scale (C-TSE) to evaluate in-service elementary school teachers, demonstrating its reliability and validity. Their studies also revealed that teacher efficacy varied by gender, teaching experience, and school type—for example, female teachers reported higher efficacy, while those in direct subsidy schools showed lower levels.

### 1.3. The Current Study

Most research on teacher self-efficacy measurement has concentrated on primary and secondary education (Skaalvik & Skaalvik, 2014; Tschannen-Moran & Hoy, 2007; Sun & Yin, 2025), while studies on preschool teachers’ self-efficacy remain underexplored (W. T. Chan et al., 2024; Steigleder et al., 2023). Although similar mechanisms may underline self-efficacy across educational levels (Sun & Yin, 2025), preschool education’s distinct characteristics —developmentally appropriate practices (NAEYC et al., 2022), game-based learning (Smidt, 2011), high levels of emotional support (Pianta et al., 2008), and intensive teacher–child interactions (Thijs et al., 2011)—may limit the generalizability of mainstream measurement tools like the OSTES. Previous research has indicated that both the structure of teacher self-efficacy and its association with child development outcomes may differ significantly between preschool and primary/secondary educational settings (Guo et al., 2010). According to Bandura (1997, 2006), self-efficacy is a domain-specific construct specific and measurement tools must align with the instructional context (Usher & Pajares, 2008). As an important aspect of professional identity (Beijaard et al., 2004), the conceptual connotation of preschool teacher self-efficacy should fully reflect its professional characteristics. Moreover, cultural differences must not be overlooked. Local contextual factors—such as parent–school expectation conflicts in collectivist cultures, large class management, and policies that integrate caregiving with education (Jiang et al., 2021)—may lead to a self-efficacy structure among Chinese preschool teachers that deviates from Western theoretical frameworks and measurement tools.

To advance research in this area and support early childhood educators in China, it is essential to develop a self-efficacy measurement tool that reflects both cultural relevance and domain specificity. Although the OSTES has been adapted for pre-service preschool teachers and in-service elementary teachers in China (e.g., H. Cheung, 2006; H. Y. Cheung, 2008; Tsui & Kennedy, 2009; Lu et al., 2020; Shao et al., 2025), its psychometric validity and contextual relevance for in-service preschool teachers—who navigate distinct challenges such as large class sizes, intensive home–kindergarten collaboration, and play-based curriculum demands—remain underexplored. This study, therefore, specifically targets a large-scale sample of in-service preschool teachers across multiple provinces to fill this gap, ensuring the SCPTSE captures the unique instructional, management, and engagement tasks of in-service preschool teachers in China. Therefore, this study sets out two primary objectives:

(1) To localize the OSTES framework and develop a culturally adapted Self-Efficacy Scale for Chinese Preschool Teachers, tailored to the specific working context, roles, and responsibilities of in-service preschool teachers in China.
(2) To rigorously validate the SCPTSE’s reliability and construct validity using both Classical Test Theory and Item Response Theory methodologies.

## 2. Materials and Methods

### 2.1. Participants

Following the principles of convenience and inclusivity, questionnaires were distributed through the Wenjuanxing (https://www.wjx.cn/) platform to invite preschool teachers in three provinces (Zhejiang, Henan, and Shaanxi) to participate voluntarily and anonymously. At the beginning of the survey, educators were presented with a preamble outlining the study’s intent, inclusion/exclusion criteria, and assurances of confidentiality. Ethical approval was obtained from the Ethics Committee of Zhejiang Normal University (IRB No. ZSRT2025113), Jinhua, Zhejiang, China. In addition to obtaining informed consent, we anonymized all data, stored responses on a secure server. Individual-level results were not returned to avoid potential misinterpretation or undue influence, but participants were invited to request further clarification if desired. Inclusion criteria required participants to be full-time preschool teachers with at least three months of continuous classroom teaching experience; exclusion criteria ruled out part-time teachers, administrative staff without regular classroom duties, and those on extended leave during data collection. This study adopted a combination of stratified and random sampling methods: beyond province economic level, sampling strata comprised kindergarten location and classroom type. Teachers from 164 kindergartens in cities such as Hangzhou, Shaoxing, and Quzhou in the Zhejiang Province; Zhengzhou, Kaifeng, and Puyang in the Henan Province; and Xi’an, Baoji, and Ankang in the Shaanxi Province were selected as survey subjects. A total of 882 valid responses were obtained. The average age of preschool teachers was 30.41 years. The comprehensive details of the participants are provided in Table 1.

### 2.2. Translation and Adaptation

To develop the self-efficacy scale for Chinese in-service preschool teachers, this study drew upon the original long form of the OSTES (Tschannen-Moran & Hoy, 2001) and followed standardized four-step cross-cultural adaptation procedure proposed by Beaton et al. (2000). This approach ensured that the newly constructed instrument retained the core evaluative framework of OSTES while aligning closely with the professional responsibilities and everyday practices of Chinese preschool teachers. The adaptation process consisted of the following steps:

Step 1: Forward Translation

Two bilingual doctoral students in education independently translated the OSTES items from English into Chinese. The translation prioritized semantic accuracy while adapting terminology to fit the context of early childhood education in China. For instance, “students” was translated as “幼儿 (children)”, and “school” was adapted to “幼儿园 (kindergarten)” to match the age group and institutional setting. “Disruptive behavior” was translated as “不当行为 (inappropriate behavior)” to avoid the negative connotation associated with “destructive” within Chinese educational ethics. Additionally, “schoolwork” was rephrased as “游戏活动 (play-based activities)”, reflecting national guidelines that emphasize play as the fundamental learning activity in preschool education (Ministry of Education of the People’s Republic of China, 2001, 2022).

Step 2: Item Adaptation and Reconstruction

While retaining the original OSTES’s three-dimensional framework—instructional strategies, classroom management, and child engagement—the researchers systematically adapted and reconstructed the 24 items to suit the Chinese preschool educational contexts. In this process, the recommendations by Bandura (2006) for constructing teacher self-efficacy scale items were closely followed: (a) each item should include verbs like “can” or “be able to” to capture perceived capability; (b) the subject in each statement should be “I” to directly assess the teacher’s capability; and (c) each item should incorporate a barrier, because if there are no obstacles to overcome, the task is inherently easy, leading to uniformly high efficacy ratings. Thus, constructing a validity self-efficacy scale requires clear conceptualization of both performance determinants in the relevant domain and the potential impediments (Bandura, 2011b).

For self-efficacy for instructional strategies, the study drew upon the 13 effective teaching strategies for preschool teachers proposed by NAEYC et al. (2022)—including encouragement, recognition, promoting peer learning, demonstration, modeling, providing clues or hints, delivering information, presenting new content, giving instructions, creating moderate challenges, offering multiple ways to support active inquiry about conceptual understanding, and providing specific feedback. Accordingly, preschool teachers’ self-efficacy in teaching was refined into four core components: (1) encouragement and recognition, (2) demonstration and guidance, (3) questioning and feedback, and (4) observation and assessment. For instance, the original item “To what extent can you use a variety of assessment strategies” was adapted to “I can flexibly employ a variety of methods to objectively assess children’s developmental status”. New items were also added, such as “I can provide clues or hints to enable children to gain a deeper understanding of new content” and “I can observe and record situations where children exhibit interest or encounter meaningful challenges” (Ministry of Education of the People’s Republic of China, 2022).

For self-efficacy for classroom management, the study utilized the Classroom Organization framework proposed by Pianta et al. (2008), which comprises three dimensions: behavior management, productivity, and instructional learning formats. Preschool teachers’ self-efficacy for classroom management was thus refined into three key aspects: behavior management, classroom efficiency, and instructional learning arrangements. Accordingly, the original item “How much can you do to control disruptive behavior in the classroom?” was adapted to “I am capable of effectively reducing inappropriate behavior among children in the classroom or using subtle cues to correct such behavior”. In addition, new items were introduced, such as “I can arrange learning or activity schedules based on children’s interests”.

For self-efficacy for child engagement, the adaptation focused on aligning the original items with the preschool context. For instance, the original item “How much can you do to help your children think critically?” was adapted to “I am capable of effectively promoting the development of critical thinking in children”, and “How much can you do to foster child creativity?” became “I am capable of effectively promoting the development of creativity in children”.

After this round of adaptation and reconstruction, an initial version of the PSESPT—comprising three dimensions and a total of 24 items—was formed.

Step 3: Expert Review and Item Optimization

A panel of experts, including three researchers in the field of preschool education and two experienced kindergarten principals, evaluated the initial items over multiple review rounds. They focused on assessing the clarity of expression, the degree of situational embedding, and cultural adaptability to ensure strong content validity for the SCPTSE (Tsui & Kennedy, 2009). The expert panel recommended two key revisions: (a) modifying items with similar content, and (b) emphasizing that Chinese preschool teachers must address child engagement at three levels—enabling children’s engagement in decision-making, engagement in the design or process of activities, and balancing individual versus group engagement.

Based on this feedback, the researchers systematically optimized the items. For instance, items like “I can use multiple methods to support children in actively understanding and applying concepts” and “I can provide targeted instructions in advance to guide their actions and behaviors” were removed. The items under efficacy for child engagement were rewritten into six refined items, such as “I can allow children to choose play materials, peers, and methods independently” and “I can organize group activities for all children while also permitting some children to choose and participate in suitable activities according to their own preferences”. After this round of review and optimization, the PSESPT was finalized with three dimensions and a total of 21 items.

Step 4: Pilot Testing

A pilot test was conducted with 15 preschool teachers in Zhejiang Province. Open-ended questions were used to collect feedback on the understandability and situational appropriateness of the items. Based on the teachers’ responses, minor revisions were made. For example, the item “I can predict and effectively prevent the occurrence or escalation of inappropriate behavior among children, thereby avoiding disruption to curriculum implementation” was modified to “I can predict and effectively prevent the occurrence or escalation of inappropriate behavior among children, thereby avoiding disruption to activity implementation”.

Through this rigorous and systematic cross-cultural adaptation process, the study ultimately developed a trial version of the PSESPT. This instrument retains the core structure of the OSTES and integrates unique cultural elements and practical demands specific to Chinese preschool education. The next phase will involve large-scale data collection to further examine its psychometric properties.

### 2.3. Measures

#### 2.3.1. Demographic Characteristics

Demographic measures included province (Zhejiang, Henan, or Shaanxi), kindergarten location (urban center, county seat, rural area), classroom type (classroom for 3–4-year-olds, classroom for 4–5-year-olds, classroom for 5–6-year-olds), years of teaching experience (3 years and below, 4–8 years, 9–14 years, or 15 years and above), teaching certification (no teaching certification, early childhood teaching certification, primary and secondary school teaching certification, or other teaching certifications), professional title (junior/unclassified, intermediate, associate senior, or full senior), highest education level (high school/vocational school or below, associate degree, bachelor’s degree, or postgraduate), and highest education level (early childhood education or early childhood education, education, or other majors).

#### 2.3.2. Scale for Chinese Preschool Teachers’ Self-Efficacy (SCPTSE)

The Scale for Chinese Preschool Teachers’ Self-Efficacy (SCPTSE) was developed based on the Ohio State Teacher Efficacy Scale (OSTES) originally proposed by Tschannen-Moran and Hoy (2001). The complete set of items used in this study is provided in Appendix A.1 (final Chinese version of the SCPTSE), Appendix A.2 (English translation of the SCPTSE), and Appendix A.3 (original OSTES-24; not formally validated). The SCPTSE is specifically tailored to the Chinese preschool education context and comprises three dimensions: self-efficacy for instructional strategies, classroom management, and child engagement, encompassing a total of 21 items. An example item from the scale is: “I can flexibly apply various methods to objectively assess the developmental status of children.” Respondents rated their perceived level of self-efficacy using a 5-point Likert scale, ranging from 1 (strongly disagree) to 5 (strongly agree), with higher scores indicating a stronger sense of self-efficacy.

### 2.4. Data Analysis

Data were analyzed using IBM SPSS Statistics version 26 and R Studio for Mac (Version 4.4.1). Specific R packages used for each analytic procedure are reported within the respective subsections. The psychometric properties of the SCPTSE were evaluated through a combined approach incorporating both CTT and IRT frameworks.

First, data normality was assessed by calculating skewness and kurtosis. Following Kim (2013), data were considered to meet the assumption of normality when the absolute value of skewness and kurtosis were below 2 and 7, respectively.

Under the CTT framework, internal consistency reliability was examined using item-total correlations (ITC) and Cronbach’s alpha (α). According to Nunnally and Bernstein (1994), items with ITC values below 0.40 and lacking statistical significance were considered to demonstrate poor homogeneity. To assess the overall internal consistency of the instrument, Cronbach’s alpha coefficients were calculated. A Cronbach’s alpha value exceeding 0.70 was interpreted as acceptable, based on the criteria proposed by Tavakol and Dennick (2011).

Subsequently, construct validity was examined using Confirmatory Factor Analysis (CFA). Prior to conducting CFA, the Kaiser–Meyer–Olkin (KMO) test and Bartlett’s test of sphericity were used to evaluate the appropriateness of the data for factor analysis, with KMO values above 0.80 indicating adequacy for factor analysis. CFA was then conducted to assess the extent to which the hypothesized factor structure of the SCPTSE was supported by the empirical data. Model fit was evaluated using multiple indices following the recommendations of Hu and Bentler (1999) and Schermelleh-Engel et al. (2003):

(a) Absolute fit indices included the chi-square to degrees of freedom ratio (χ^2^/df), with values below 5 considered acceptable and below 3 indicating good fit. Root Mean Square Error of Approximation (RMSEA) values ≤ 0.08 were interpreted as reasonable, and ≤0.06 as good. Standardized Root Mean Square Residual (SRMR) values < 0.08 were considered indicative of good model fit.
(b) Relative fit indices included the Comparative Fit Index (CFI), Tucker–Lewis Index (TLI), Normed Fit Index (NFI), and Incremental Fit Index (IFI). CFI and TLI values > 0.85 indicated acceptable fit, and values > 0.90 indicated good fit. NFI values > 0.80 indicated acceptable fit. IFI values > 0.90 indicated acceptable fit.
(c) Parsimony fit indices, including the Parsimonious Normed Fit Index (PNFI) and the Parsimonious Goodness of Fit Index (PGFI), were used to evaluate the balance between model complexity and explanatory power. Values > 0.50 were interpreted as indicative of satisfactory model parsimony.

For the IRT-based analyses, given the polytomous nature of the SCPTSE and the ordered structure of its response categories, the Graded Response Model (GRM) was adopted (Samejima, 1997). Before estimating model parameters, two key assumptions were tested: (a) unidimensionality, which assumes that items measure a single latent trait (Yang & Kao, 2014), and was assessed by Loevinger’s H coefficients (Loevinger, 1947) for each three subscales. Loevinger’s H coefficients between 0.3 and 0.4 are considered as weakly unidimensional, H coefficients between 0.4 and 0.5 as moderately unidimensional, and H coefficients above 0.50 as strongly unidimensional (Sijtsma & Molenaar, 2002), and (b) local independence, evaluated using Yen’s Q_3_ residual coefficient (Yen, 1984) among items of each subscale. We adopt the more lenient criterion for Q3 recommended by Christensen et al. (2016), setting the threshold at less than |0.30| (e.g., Ramp et al., 2009). This approach reduces false-positive detections caused by minor local dependencies and is more appropriate for preserving content validity in practical scale development.

Upon confirming these assumptions, the two-parameter logistic (2PL) model was employed to estimate item discrimination (*a*) and difficulty (*b*) parameters. According to Baker and Kim (2017), *a*-parameters were classified as very low (0.01–0.34), low (0.34–0.64), moderate (0.65–1.34), high (1.35–1.69), and very high (>1.70). The *b*-parameters, indicating item difficulty thresholds, were expected to fall within the range of −4 to +4. Additionally, Item Information Function (IIF) and Test Information Function (TIF) were computed to assess the measurement precision of each item and the overall scale, respectively. Following Jiangxi Normal University “Modern Educational and Psychometric General Analysis System” Development Group et al. (2003), a test-level information value ≥ 25 was considered excellent, values between 16 and 25 indicated moderate quality, and values < 16 reflected poor measurement precision. For 5-point Likert scales, item-level information quality was determined by calculating the average IIF value across the θ range from −4 to +4 in 0.1 intervals. According to Tu and Cai (2005), the average IIF value of items below the threshold value (based on the formula 25 × 5/number of items/5) was flagged as underperforming and considered for revision or deletion.

## 3. Results

### 3.1. Descriptive Statistics and Partial Correlation Analysis

As shown in Table 2, the overall level of the CPTSE had a mean score of 3.892 (SD = 0.692). The scores across dimensions were relatively close: IS (M = 3.968, SD = 0.710), CM (M = 3.823, SD = 0.715), and SE (M = 3.886, SD = 0.746), indicating that the surveyed preschool teacher group exhibited relatively stable self-efficacy. After controlling for variables such as teaching experience, qualifications, professional titles, and educational background, partial correlation analysis revealed significant positive correlations (*p* < 0.001) between CPTSE and all three dimensions: a correlation coefficient of 0.950 with IS, 0.959 with CM, and 0.952 with SE. This demonstrates that preschool teachers’ self-efficacy in instructional strategies, classroom management, and child engagement significantly contributes to their overall self-efficacy. Notably, high covariant relationships were observed among the dimensions (IS-CM r = 0.876, IS-SE r = 0.853, CM-SE r = 0.877), which validates the theoretical rationality of the scale construction and suggests potential conceptual overlaps requiring attention. Furthermore, skewness (ranging between −0.445 and −0.157) and kurtosis values (ranging between −0.171 and 0.410) fell within the thresholds suggested by Kim (2013) for normality, further confirming the normal distribution of data suitable for subsequent statistical analyses.

### 3.2. CTT Analysis

As shown in Table 3, all 21 items of the CPTSE exhibited significant correlations (*p* < 0.001) with the total score, with coefficients ranging from 0.806 to 0.890, each exceeding 0.80. This indicates that each item robustly reflects the overall level of preschool teachers’ self-efficacy, reflecting the scale’s representativeness and stability while contributing evenly across items. Table 2 further shows a Cronbach’s alpha of 0.992 for the full scale, indicating excellent internal consistency. The Cronbach’s alpha coefficients for the individual dimensions range between 0.950 and 0.959, each well above the acceptable threshold of 0.90, thereby confirming consistency at both scale and sub-scale levels. Furthermore, deleting any item did not substantially improve alpha values, further supporting the scale’s reliability.

To assess the structural validity of the SCPTSE, three competing models were tested: a one-factor model (Model 1), an optimized model (Model 2) with additional paths indicated by the maximum modification indices (MI), and a theoretically driven three-factor model (Model 3). The data were appropriate for factor analysis, as indicated by a KMO value of 0.980 and a significant Bartlett’s test, χ^2^ (210) = 21,603.576 (*p* < 0.001).

Table 4 presents the fit indices for all three models, with the path diagram of the three-factor model supporting the structure’s validity. In terms of absolute fit indices, the one-factor model (Model 1) exhibited an RMSEA of 0.115, which is considerably above the acceptable threshold of 0.08, indicating poor fit. Both the modified Model 2 and the three-factor model (Model 3) achieved RMSEA values within the acceptable range (<0.08, each at 0.079). Additionally, the three-factor model had the lowest SRMR (0.025), outperforming Model 2 (0.028) and Model 1 (0.038), indicating the best fit at the residual level. Among the three models, the three-factor model yielded the lowest chi-square to degrees of freedom ratio (χ^2^/df = 6.45, *p* < 0.001). Although slightly above the ideal threshold (<5), this index is highly sensitive to large sample sizes and should be interpreted with other fit indices (Alavi et al., 2020). In terms of relative fit indices, Model 1’s Comparative Fit Index (CFI = 0.898) and Tucker–Lewis Index (TLI = 0.886) fell just below 0.90. In contrast, Models 2 and 3 showed significant improvements, with CFI/TLI values of 0.955/0.947 and 0.953/0.947, respectively. These minor differences suggest that both models possess strong relative fit. Additionally, Model 3 performed slightly better in the Normed Fit Index (NFI = 0.945) and Incremental Fit Index (IFI = 0.953) compared to Model 2 (NFI = 0.947, IFI = 0.955) and Model 1 (NFI = 0.890, IFI = 0.898), supporting the theoretical strength of the three-factor structure. With respect to parsimonious fit indices, the three-factor model’s PNFI (0.837) and PGFI (0.700) were higher than those of Model 2 (0.807, 0.671) and Model 1 (0.801, 0.607), indicating that the three-factor model better balances complexity and model fit, demonstrating robust parsimony and stability.

**Figure 1:**
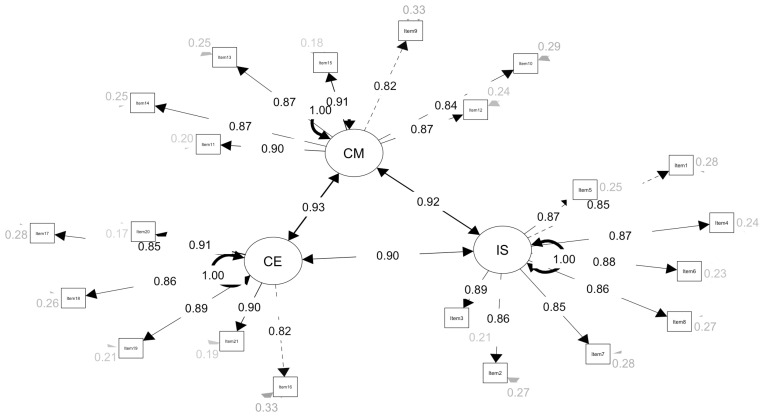
Factor Structure of Model 3 (three-factor model) for the SCPTSE, with standardized parameters. *Note*. IS: self-efficacy for instructional strategy; CM: self-efficacy for classroom management; CE: self-efficacy for child engagement.

Furthermore, the path diagram for the three-factor model (Figure 1) reveals that all standardized factor loadings of all measurement items on their respective latent variables exceed 0.80, confirming strong convergent validity for each sub-scale. Correlations among the three latent factors ranged from 0.90 to 0.93, indicating strong interrelationships while retaining structural distinctiveness, further supporting the model’s robust structural validity.

Overall, the three-factor model exhibits superior performance across absolute, relative, and parsimonious fit indices. It scientifically reflects the relationship between the theoretical constructs and the empirical data, confirming SCPTSE’s strong structural validity.

### 3.3. IRT Analysis

First, based on the results presented in Table 2, all three subscales demonstrated strong unidimensionality, as evidenced by Loevinger’s H coefficients exceeding 0.80 (H_IS_ = 0.812, H_CM_ = 0.800, H_CE_ = 0.818). These findings provide robust psychometric support for treating each subscale as a distinct and coherent latent construct, thereby justifying the use of unidimensional item response models for subsequent analyses.

**Figure 2:**
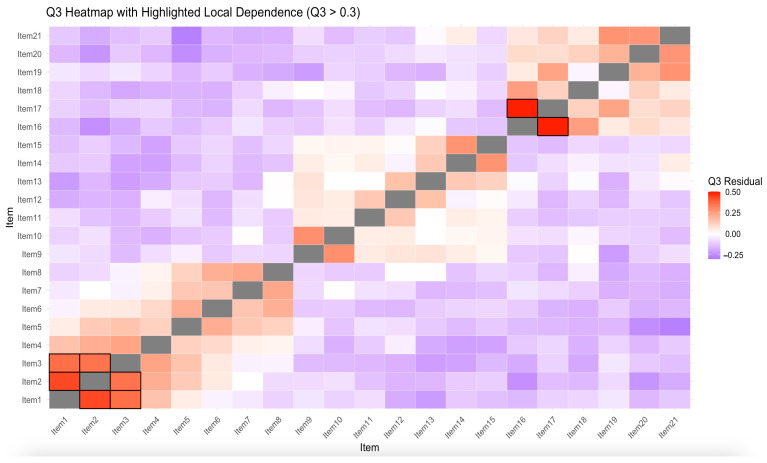
Q3 heatmap with highlighted local dependence. *Note.* Item pairs with Q3 values greater than 0.3 are highlighted with bold-bordered red cells, indicating potential local dependence.

Second, in the local independence test, Figure 2 displays the residual correlations (Q_3_ values) among item pairs. Warmer colors (red) indicate higher residual correlations, while cooler colors (purple) represent lower or negative correlations. The figure shows that most item pairs in the SCPTSE have Yen’s Q_3_ values below |0.30|. A few pairs—Item 1 with Items 2 and 3 (Q_3_ = 0.431, 0.362), Item 2 with Item 3 (Q_3_ = 0.350), and Item 16 with Item 17 (Q_3_ = 0.497)—show Q_3_ values exceeding the threshold. Although these signals of local dependency exist, none of the Q_3_ values reach the level of strong dependence (|Q_3_| > 0.50) (Christensen et al., 2016), which indicates that the impact of these dependencies on the measurement model is limited. Overall, the SCPTSE still meets the unidimensionality requirement and is suitable for further analysis.

Finally, Table 3 reports the discrimination parameters (a) for the 21 items, four level difficulty parameters (*b*_1_, *b*_2_, *b*_3_, *b*_4_), and the item information function mean value of each item (IIFM). Figure 2 displays the test information function curve for the ability interval [−4, 4]. In the discrimination analysis, the mean of *a*-parameter across the 21 items is approximately 3.557. All items have discrimination parameters exceeding the commonly acceptable standard (all > 1.7), reaching an excellent level, indicating these items are highly effective in distinguishing varying levels of self-efficacy among preschool teachers. The difficulty parameters fall within the expected range of [−4, 4]. Specifically, the average values of the difficulty parameters are as follows: *b*_1_ = −3.443 (range: −3.815 to −3.003), *b*_2_ = −2.334 (range: −2.590 to −2.037), *b*_3_ = −0.503 (range: −0.828 to −0.295), and *b*_4_ = 0.771 (range: 0.316 to 1.155). Although the overall difficulty is relatively low, particularly for *b*_1_, the consistently increasing pattern (*b*_1_ < *b*_2_ < *b*_3_ < *b*_4_) indicates a well-structured design that effectively differentiates performance across various ability levels.

In terms of information functions, the total test information function is 33.580—well above the ideal threshold of 25. Based on Tu and Cai’s (2005) criteria, items with average information value exceeding 1.190 are considered good, while those between 0.761 and 1.190 need improvement (Tu & Cai, 2005). As presented in Table 3, the mean item information function values for the 21 items range between 1.036 and 2.148. Apart from Item 16, the other 20 items have mean item information function values above the good standard, indicating that these 20 items can provide sufficient information across different levels of the latent trait, effectively distinguishing teachers with varying levels of self-efficacy and accurately reflecting their self-efficacy performance. However, Item 16 falls below the desired level and may require further revision to enhance its psychometric performance.

In summary, the SCPTSE demonstrates excellent discrimination capabilities, overall reasonable difficulty, and balanced information distribution. It can provide accurate and reliable measurement across a broad spectrum of trait levels, which is conducive to identifying differences in self-efficacy among teachers. Nevertheless, the relatively low-difficulty design—especially at the lower level (e.g., *b*_1_)—indicates room for improvement.

## 4. Discussion

Through a systematic cross-cultural adaptation process, this study integrated the core structure of the OSTES (Tschannen-Moran & Hoy, 2001) with the cultural and practical context of Chinese preschool education to develop the SCPTSE. Employing a combination of CTT and IRT, the study systematically examined the scale’s psychometric properties within the context of Chinese preschool education. Results show that the SCPTSE demonstrates high internal consistency, strong structural validity, and robust item parameters. Compared with Shao et al. (2025), this study further confirms the SCPTSE’s effectiveness in measuring the self-efficacy of in-service Chinese preschool teachers across instructional strategies, classroom management, and child engagement. These findings provide new empirical support and methodological insights for localizing and extending the OSTES in non-Western contexts.

### 4.1. Cultural Adaptability of the SCPTSE: Integrating Policy Orientation and Local Practices

Teacher self-efficacy is a multidimensional and dynamic psychological construct shaped by individual factors and cultural traditions, educational systems, and policy orientations (Bandura, 1997; Tschannen-Moran & Hoy, 2001). Therefore, adapting self-efficacy measures requires both rigorous psychometric standards and cultural ecological relevance (Hambleton & Zenisky, 2011). The development of the SCPTSE reflects this approach, demonstrating strong cultural adaptability through its alignment with the ecological realities, policy orientations, and professional roles within Chinese preschool education.

First, the item design aligns closely with the policy directives and practical scenarios of Chinese preschool education. For instance, Item 19—“I can allow children to choose play materials, peers, and methods independently”—highlights the longstanding emphasis on “play as the fundamental activity” in preschool education in China and echoes recent discussions on the balance between teacher guidance and child autonomy in play-based learning (Yin et al., 2022). Similarly, Item 17—“I can involve children in decisions related to their daily lives”—reflects the core requirements of China’s integrated care and education policy (Jiang et al., 2021) and embodies the dynamic tension that teachers must navigate between institutional norms and child autonomy in a collectivist culture (K. Li, 2024).

Second, the items are closely aligned with the professional responsibilities of Chinese preschool teachers, who manage instructional tasks but also large class sizes and high emotional labor (Zhou et al., 2020; X. Li et al., 2021). In response, the SCPTSE reconstructed several OSTES items to reflect these contextual demands. For instance, Item 7—“I can observe and record situations where children exhibit interest or encounter meaningful challenges”—strengthens the role of teachers in the daily “observation and documentation” required in classroom practice. Item 8—“I can flexibly employ a variety of methods to objectively assess children’s developmental status”—reflects the recent policy shifts toward comprehensive, developmental evaluation in preschool education in China (Ministry of Education of the People’s Republic of China, 2022), thereby integrating “holistic, ongoing assessments” into teachers’ responsibilities. Moreover, Item 21—“I can organize group activities for all children while also permitting some children to choose and participate in suitable activities according to their own preferences”—and Item 20—“I can fully consider the learning and development needs of group children and the special needs of individual children”—capture the deep-seated educational practice demands in recent years. In a collectivist culture, it is essential to maintain a relative balance between collective and individual needs (K. Li, 2024). Unlike Western tools such as CLASS (Pianta et al., 2008), which emphasize individual autonomy, the SCPTSE highlights teachers’ dual responsibility: maintaining order within a structured environment while supporting individual development.

In short, this study not only provides robust empirical evidence for the structural and psychometric soundness of the SCPTSE but also illustrates how educational measurements can be thoughtfully tailored to reflect local cultural and policy environments.

### 4.2. SCPTSE’s Psychometric Properties: Multi-Method Validation and Precision Optimization

Developing assessment tools for preschool teachers requires both professional rigor and methodological complexity. Adhering to a robust psychometric process is essential to ensure scientific validity and practical relevance. The SCPTSE was thoroughly evaluated using both Classical Test Theory (CTT) and Item Response Theory (IRT). CTT analyses confirmed that the SCPTSE possesses high internal consistency and strong structural validity, while IRT offered detailed item-level insights, demonstrating the scale’s measurement precision and identifying potential areas for future refinement.

In the CTT analysis, this study not only assessed the internal consistency of the SCPTSE but also compared the goodness-of-fit of unidimensional, revised, and three-factor theoretical models through Confirmatory Factor Analysis (CFA). The overall Cronbach’s α was 0.980, with subscale α coefficients ranging from 0.950 to 0.959—exceeding those of the original scale (Tschannen-Moran & Hoy, 2001) and those reported in studies conducted in cultural contexts such as Mexico (Salas-Rodríguez et al., 2021) and Hong Kong (Tsui & Kennedy, 2009).

For structural validity, the SCPTSE’s three-factor model (instructional strategies, classroom management, and child engagement) demonstrated superior fit compared to the one-factor model, thereby confirming the validity of the theoretical construct originally proposed by Tschannen-Moran and Hoy (2001) for in-service teachers. This finding aligns with evidence from various countries, including China (Shao et al., 2025; Lu et al., 2020; Tsui & Kennedy, 2009), Canada, Cyprus, Korea, Singapore, and the United States (Klassen et al., 2009), Greece (Tsigilis et al., 2010), and Mexico (Salas-Rodríguez et al., 2021), collectively supporting the cross-cultural stability of a three-dimensional self-efficacy across cultures.

This study further confirms teacher self-efficacy among in-service preschool teachers in China. Notably, Ruan et al. (2015) suggested that the 24-item long form of the TSES did not fit Chinese data well, with the 12-item short form performing better. In contrast, our findings indicate that the long form SCPTSE is highly applicable to preschool teachers in mainland China. Moreover, the SCPTSE outperformed many other studies in several fit indices (e.g., RMSEA, CFI, TLI, SRMR). For instance, Klassen et al. (2009) found RMSEA values exceeding the critical threshold for teachers in Cyprus (0.105) and Korea (0.134); Valls et al. (2020) reported a high RMSEA (0.097) and low TLI (0.87) among Swiss teachers; similarly, Salas-Rodríguez et al. (2021) documented an RMSEA of 0.102 and a TLI of 0.886 in a sample of Mexican teachers. These strong results may be attributed to the study’s rigorous cross-cultural adaptation process, based on Beaton et al.’s (2000) four-stage method—forward translation, item adaptation and restructuring, expert review and item optimization, and pilot testing—which ensured the scale’s alignment with the practical realities of Chinese early childhood education.

Another noteworthy finding is that, despite the high inter-factor covariances (φ = 0.897–0.927), model comparisons continue to support the independence of the three-factor structure. This observation can be interpreted from both cultural and professional perspectives: the integrative characteristics of teacher self-efficacy are more pronounced in Chinese culture, where a Confucian tradition of holistic thinking views professional competencies as an organic whole rather than a collection of discrete skills (J. Li, 2012). This characteristic reflects, to some extent, the intrinsic interplay among various tasks in a teacher’s actual work (Pan et al., 2024). Nonetheless, such high inter-factor covariances also suggest that these subdimensions may operate synergistically. Accordingly, future research should examine a higher-order general self-efficacy factor or apply bifactor modeling to disentangle domain-general from domain-specific variance. Moreover, future research may examine item redundancy to develop a shorter form suitable for preschool settings. Qualitative investigations—such as in-depth interviews or focus groups with in-service preschool teachers—could further illuminate how educators perceive and enact these interrelated tasks in practice. Supporting this integrative view, Tsui and Kennedy (2009) found that Hong Kong teachers did not differentiate between self-efficacy for student engagement and instructional strategies; instead, these items merged into a unified construct of teaching and support (TS). This suggests that educators may perceive teaching and student engagement as intertwined and mutually reinforcing aspects of professional practice.

Regarding IRT, although several cross-cultural studies based on the OSTES have been conducted, most have not employed IRT methods to delve deeply into the psychometric properties at the item level. This study introduced IRT analysis for the SCPTSE, systematically revealing the scale’s advantages in measurement precision as well as identifying areas for improvement. First, all items demonstrated discrimination parameters exceeding 1.70—classified as “excellent”— which suggests the scale is highly effective in differentiating between teachers with varying levels of self-efficacy. This strength may be related to the characteristics of efficacy expression in Chinese culture; the use of first-person constructions (i.e., “I can…”) (Bandura, 2006) is more likely to evoke embodied cognition and situational empathy, prompting teachers to engage in reflective self-assessment based on concrete behaviors and contextual details.

**Figure 3:**
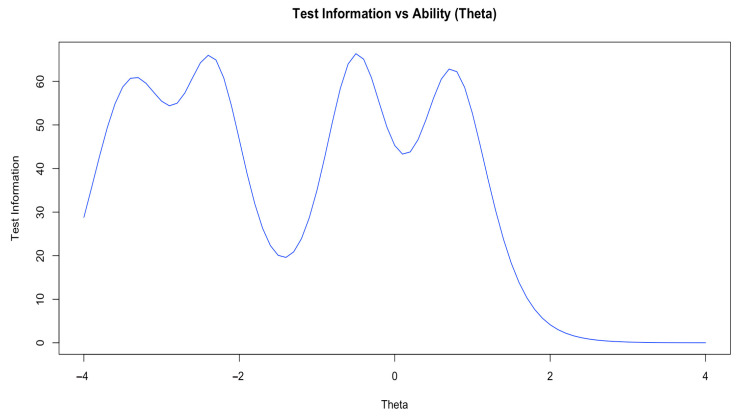
Test information function for the SCPTSE.

Secondly, concerning the item difficulty parameters (*b*_1_–*b*_4_), the stepwise progression across levels aligns with theoretical expectations, suggesting that the SCPTSE has a well-established gradient of difficulty. However, the low difficulty parameter (*b*_1_) suggests that some items may be too easy, leading to an “easy-to-answer” phenomenon. As shown in Figure 3, analysis of the test information function revealed multiple peaks in the ability range of θ = −3.5 to θ = 1.0, which provides precise measurement for teachers with low to moderate self-efficacy levels. Conversely, when θ > 2.0, the information function drops rapidly below 10, signaling a “ceiling effect” with insufficient discrimination among high-ability teachers This phenomenon may be attributable to two factors: on the one hand, the structural limitation of the scoring system, as the SCPTSE employs a 5-point rating scale that compresses variance at the high-ability end; in contrast, other studies often use 9-point scales to enhance resolution among high achievers (e.g., Shao et al., 2025; Tsui & Kennedy, 2009; Ruan et al., 2015). On the other hand, the ceiling effect may also reflect social desirability bias in self-reporting, as evidenced by the generally elevated scores across both the overall scale and its subdimensions (Paulhus, 2002). This upper limit effect exhibits cross-cultural similarities, as noted in Mexican teacher samples (Salas-Rodríguez et al., 2021). Based on these findings, two recommendations for future revisions of the SCPTSE are proposed. First, more challenging items or complex scenarios should be introduced to increase item difficulty and meet the progressive demands of teacher professionalism in global early childhood education (NAEYC et al., 2022; Yin et al., 2022). Simultaneously, incorporating reverse-worded items could help reduce response bias. Second, the scoring system should be optimized by expanding the current 5-point rating scale to a 7- or 9-point scale. Prior research (K. Li et al., 2014) shows that increasing the number of rating levels improves measurement sensitivity and precision, addressing the structural and discrimination limitations of the original scale.

Finally, it is noteworthy that Item 16 (“I can involve children in discussing and formulating class rules”) displayed an average Item Information Function Mean (IIFM = 1.036) below the acceptable standard of 1.190, indicating a deficiency in its measurement focus. This may be due to the inherent complexity of the behavioral target and situational ambiguity in the item’s phrasing. The phrase “discussing and formulating” involves multiple sub-behaviors, such as proposing rules, negotiating, and building consensus, and the lack of specification regarding the context for rule formulation may lead to varied interpretations and judgments among respondents. This variability weakens the item’s measurement precision and internal consistency. Future revisions could address this by disaggregating the item or providing more context to enhance its measurement focus and discriminative capacity (DeVellis, 2016).

### 4.3. Theoretical and Practical Significance

The results of this study have significant theoretical and practical implications. From a theoretical standpoint, the SCPTSE makes notable contributions. While it retains the stable three-dimensional structure of teacher self-efficacy, it has been fully adapted to the specific cultural and practical context of Chinese preschool education. In doing so, it localizes and extends the original three dimensions of the OSTES. Specifically, within the “self-efficacy for instructional strategies” dimension, it is further refined into four components: “encouragement and recognition”, “demonstration and guidance”, “questioning and feedback”, and “observation and assessment”. In the “self-efficacy for classroom management” dimension, the construct is divided into three key aspects: “behavior management”, “learning arrangements”, and “classroom productivity”. Meanwhile, the “self-efficacy for child engagement” dimension is detailed into three core elements: “supporting children’s engagement in decision-making”, “supporting children’s engagement in the process of activities”, and “balancing individual and group needs”. This refined structure not only reflects the authentic scenarios of professional teaching practice but also enriches and deepens the theoretical connotations of self-efficacy, offering new theoretical perspectives for the localization of cross-cultural measurement instruments (Tsui & Kennedy, 2009).

Secondly, through rigorous empirical testing, the study confirmed the structural stability and measurement validity of the SCPTSE’s three-factor model among in-service preschool teachers in China. This finding provides robust empirical support for extending teacher self-efficacy theory within the Chinese cultural context and offers a representative Chinese sample perspective for international cross-cultural comparisons of teacher self-efficacy. The study highlights the importance of considering local cultural uniqueness when adapting international theoretical frameworks. Through appropriate structural adjustments and content reconstruction, the instrument is made more aligned with real-world contexts, fostering a dynamic, bidirectional interplay between theory and practice. Moreover, by employing both CTT and IRT methods for detailed item-level analysis, the research clearly identifies the strengths of the current instrument as well as the items that need further optimization, thereby providing a clear empirical pathway for the future continuous revision and enhancement of the SCPTSE.

In practical terms, the development of the SCPTSE provides a highly relevant and actionable measurement tool for preschool education practice in China. The scale offers kindergarten administrators, teacher training organizations, and education policymakers a scientific and quantitative method for systematically assessing teachers’ self-efficacy in the key areas of instructional strategies, classroom management, and child engagement. This enables the early identification of specific skill gaps and the design of targeted professional learning modules. Furthermore, regional education authorities can incorporate the SCPTSE into ongoing teacher appraisal systems. This would allow data-driven allocation of resources more effectively, while policymakers can design incentive structures tied to demonstrated preschool teachers’ self-efficacy improvements. Importantly, the SCPTSE development process rigorously followed cross-cultural adaptation procedures. Its comprehensive process—from translation and cultural reconstruction to empirical validation—offers an instructive, practical paradigm and methodological reference for other countries and regions aiming to localize teacher self-efficacy measurement tools.

## 5. Limitations and Future Directions

Despite the satisfactory results, several limitations remain. First, the sample was primarily drawn from Zhejiang, Henan, and Shaanxi provinces, limiting national representativeness due to regional cultural differences and uneven educational resources. Future studies should include preschool teachers from central, western, and rural areas to improve generalizability. Second, relying solely on self-report questionnaires may introduce social desirability and subjective bias. Incorporating methods such as classroom observations, interviews, and longitudinal designs could further validate the scale’s stability and predictive validity. Third, although both CTT and IRT were applied, some items exhibited low difficulty levels, particularly in distinguishing low-ability respondents. Future studies should consider adjusting these items or adding more discriminative ones to ensure a balanced measurement across the entire ability continuum. Finally, this study focused primarily on psychometric validation without deeply exploring how self-efficacy influences teaching outcomes, professional development, and children’s academic and socio-emotional progress. Future studies should not only broaden sample coverage but also employ long-term longitudinal designs to better understand the evolution of teacher self-efficacy and its lasting impact on preschool education quality and teacher growth.

## Figures and Tables

**Table 1:** Demographic characteristics of participants (*n* = 882).

Variable		N	%
Province	Zhejiang	260	29.48
	Henan	309	35.03
	Shaanxi	313	35.49
Kindergarten location	Urban center	475	53.85%
	County seat	270	30.61%
	Rural area	137	15.53%
	Classroom for 3–4-year-olds	261	29.59%
Classroom type	Classroom for 4–5-year-olds	297	33.67%
	Classroom for 5–6-year-olds	324	36.73%
Year of teaching experience	3 years and below	216	24.50
	4–8 years	294	33.30
	9–14 years	206	23.40
	15 years and above	166	18.80
Teaching certification	No teaching certification	24	2.70
	Early childhood teaching certification	811	92.00
	Primary and secondary school teaching certification	37	4.20
	Other teaching certifications	10	1.10
Professional title	Junior/unclassified	755	85.60
	Intermediate	114	12.93
	Associate senior	11	1.25
	Full senior	2	0.22
Highest education level	High school/vocational school or below	16	1.81
	Associate degree	167	18.93
	Bachelor’s degree	688	78.00
	Postgraduate	11	1.25
Major of highest education level	Early childhood education or early childhood education	783	88.78
	Education or psychology-related majors	42	4.76
	Other majors	57	6.46

*Note:* Percentages may not sum to exactly 100% due to rounding.

**Table 2:** Descriptive statistics, Cronbach’s alpha, Loevinger’s H coefficients, and partial correlation analysis (r values) for the SCPTSE (*n* = 882).

	M	SD	Cronbach’s Alpha	Loevinger’s H Coefficients	CPTSE	IS	CM	CE	SI	Std. Error	KI	Std. Error
CPTSE	3.892	0.692	0.980		1				−0.281	0.082	0.407	0.164
IS	3.968	0.710	0.959	0.812	0.951 ***	1			−0.445	0.082	0.410	0.164
CM	3.823	0.715	0.955	0.800	0.960 ***	0.876 ***	1		−0.157	0.082	0.175	0.164
CE	3.886	0.746	0.950	0.818	0.954 ***	0.853 ***	0.877 ***	1	−0.180	0.082	−0.171	0.164

*Note*. M: mean; SD: standard deviation; CPTSE: Chinese preschool teacher self-efficacy; IS: self-efficacy for instructional strategy; CM: self-efficacy for classroom management; CE: self-efficacy for child engagement; SI: skewness; KI: kurtosis; control variables: years of teaching experience, teaching certification, professional title, highest education level, major of highest education level; *** *p* < 0.001.

**Table 3:** Item-Scale Correlation (r), Cronbach’s Alpha If Item Deleted, IRT Difficulty, IRT Discrimination, and Item Information Function Mean (IIFM) for the SCPTSE.

Item	Item-Scale Correlation (r)	Cronbach’s Alpha If Item Deleted	IRT Discrimination	IRT Difficulty				IIFM
			a	b1	b2	b3	b4	
Item1	0.821 ***	0.980	3.277	−3.540	−2.540	−0.827	0.316	1.409
Item2	0.814 ***	0.980	3.073	−3.580	−2.590	−0.828	0.392	1.295
Item3	0.853 ***	0.979	3.660	−3.487	−2.501	−0.717	0.541	1.648
Item4	0.842 ***	0.980	3.393	−3.510	−2.439	−0.613	0.716	1.514
Item5	0.839 ***	0.980	3.405	−3.522	−2.552	−0.460	0.833	1.504
Item6	0.856 ***	0.979	3.820	−3.469	−2.518	−0.546	0.781	1.743
Item7	0.838 ***	0.980	3.285	−3.815	−2.234	−0.489	0.867	1.429
Item8	0.848 ***	0.979	3.531	−3.271	−2.114	−0.503	0.844	1.628
Item9	0.806 ***	0.980	2.894	−3.23	−2.150	−0.318	1.155	1.275
Item10	0.832 ***	0.980	3.199	−3.338	−2.299	−0.391	1.020	1.431
Item11	0.885 ***	0.979	4.463	−3.142	−2.264	−0.482	0.682	2.099
Item12	0.859 ***	0.979	3.818	−3.453	−2.334	−0.395	0.942	1.770
Item13	0.851 ***	0.979	3.594	−3.241	−2.103	−0.295	1.027	1.668
Item14	0.848 ***	0.980	3.488	−3.476	−2.406	−0.429	0.871	1.573
Item15	0.887 ***	0.979	4.694	−3.003	−2.409	−0.486	0.785	2.148
Item16	0.806 ***	0.980	2.527	−3.686	−2.037	−0.365	0.912	1.036
Item17	0.829 ***	0.980	2.889	−3.582	−2.219	−0.403	0.815	1.238
Item18	0.859 ***	0.979	3.465	−3.483	−2.293	−0.363	0.901	1.571
Item19	0.869 ***	0.979	3.871	−3.308	−2.46	−0.595	0.490	1.744
Item20	0.890 ***	0.979	4.392	−3.768	−2.357	−0.526	0.652	2.007
Item21	0.877 ***	0.979	3.969	−3.391	−2.186	−0.528	0.643	1.853

*Note*. IIFM: item information function mean; *** *p* < 0.001.

**Table 4:** Comparison of Goodness-of-Fit Indices obtained through CFA for the SCPTSE.

Model	χ^2^	χ^2^/df	RMSEA	SRMR	CFI	TLI	NFI	IFI	PNFI	PGFI
Model 1 (unidimensional)	2398.342	12.690 ***	0.115	0.038	0.898	0.886	0.890	0.898	0.801	0.607
Model 2 (unidimensional + MI)	1160.072	6.481 ***	0.079	0.028	0.955	0.947	0.947	0.955	0.807	0.671
Model 3 (three-factor CFA)	1199.701	6.450 ***	0.079	0.025	0.953	0.947	0.945	0.953	0.837	0.700

*Note*. MI: modification index; χ^2^: Chi-square; df: degrees of freedom; χ^2^/df: Chi-square to degrees of freedom ratio; RMSEA: root mean square error of approximation; SRMR: standardized root mean square residual; CFI: comparative fit index; TLI: Tucker–Lewis index; NFI: normed fit index; IFI: incremental fit index; PNFI: parsimony normed fit index; PGFI: parsimony goodness-of-fit index; *** *p* < 0.001.

## Data Availability

The data presented in this study are available on request from the corresponding author.

## Abbreviations

The following abbreviations are used in this manuscript:

SCPTSE	Scale for Chinese Preschool Teachers’ Self-Efficacy
TSE	Teacher self-efficacy
ECE	Early childhood education
GTE	General teaching efficacy
PTE	Personal teaching efficacy
TSES	Teacher Self-Efficacy Scale
OSTES	Ohio State Teacher Efficacy Scale
CPTSE	Chinese preschool teacher self-efficacy
CLASS	Classroom assessment scoring system
IS	Self-efficacy for instructional strategy
CM	Self-efficacy for classroom management
CE	Self-efficacy for child engagement
SI	Skewness
KI	Kurtosis
EFA	Exploratory factor analysis
CFA	Confirmatory factor analysis
MI	Modification index
ESEM	Exploratory structural equation modeling
ITC	Item-total correlations
KMO	Kaiser–Meyer–Olkin
χ^2^	Chi-square
df	Degrees of freedom
χ^2^/df	Chi-square to degrees of freedom ratio
RMSEA	Root mean square error of approximation
SRMR	Standardized root mean square residual
CFI	Comparative fit index
TLI	Tucker–Lewis index
NFI	Normed fit index
IFI	Incremental fit index
PNFI	Parsimonious normed fit index
PGFI	Parsimonious goodness of fit index
GRM	Graded response model
2PL	Two-parameter logistic
IIF	Item information function
TIF	Test information function
M	Mean
SD	Standard deviation
IIFM	Item information function mean

## Appendix A

### Appendix A.1

**Table A1:** Final Chinese version of the SCPTSE.

	21个项目
教学策略效能感	1. 我能鼓励幼儿的努力和坚持，认可幼儿的言行。
	2. 当幼儿感到困惑时，我能示范做事情的正确方法、解决问题的不同方式帮助其理解。
	3. 我能提供线索或提示让幼儿对新的内容获得更深的理解。
	4. 我能根据幼儿的现有水平创造或提供不同程度的挑战性，促进幼儿在原有水平上的发展。
	5. 我能运用开放性提问或启发性提问来激发幼儿的思考。
	6. 我能针对幼儿的具体行为、学习内容、学习情境给予幼儿具体的反馈。
	7. 我能通过观察发现幼儿感兴趣或有意义的问题和情境，并进行必要的记录。
	8. 我能灵活运用多种方法对幼儿的发展状况进行客观评估。
课堂管理效能感	9. 我能预测并有效防止幼儿不当行为的出现或升级，避免影响活动实施。
	10. 我能有效减少幼儿在课堂中的不当行为或使用巧妙暗示来纠正不当行为。
	11. 我能根据幼儿的兴趣进行学习安排或活动安排。
	12. 我能聚焦学习目标，运用总结、重新定位目标等策略引导幼儿的学习。
	13. 我能建立起有效的课堂管理模式来确保幼儿学习时间的最大化。
	14. 我让儿童遵守课堂规则，有效完成课堂管理任务。
	15. 我能建立起合理的常规来确保活动顺利进行和过渡。
幼儿参与效能感	16. 我让幼儿参与班级规则的商议与制定。
	17. 我让幼儿参与一日生活中与自己有关的决策。
	18. 对活动不感兴趣或能力水平较弱的幼儿，我能提高他们对活动的参与度。
	19. 我让幼儿自主选择游戏材料、同伴和玩法。
	20. 我能充分考虑群体幼儿的学习与发展需求，也能顾及个别幼儿的特殊需要。
	21.我既组织集体的活动供全体幼儿参与，也允许一些幼儿根据自己的意愿选择和参与适宜的活动。

### Appendix A.2

**Table A2:** English translation version of the SCPTSE.

	21 Items
Efficacy for instructional strategies (IS)	1. I can encourage children’s effort and perseverance and acknowledge their words and actions.
	2. When children feel confused, I can demonstrate correct methods and different problem-solving approaches to help them understand.
	3. I can provide clues or hints to enable children to gain a deeper understanding of new content.
	4. I can create or provide varying levels of challenge based on children’s current abilities to promote their development from their existing level.
	5. I can use open-ended or probing questions to stimulate children’s thinking.
	6. I can provide specific feedback to children regarding their behaviors, learning content, and learning contexts.
	7. I can observe and record situations where children exhibit interest or encounter meaningful challenges.
	8. I can flexibly employ a variety of methods to objectively assess children’s developmental status.
Efficacy for classroom management (CM)	9. I can predict and effectively prevent the occurrence or escalation of inappropriate behavior among children, thereby avoiding disruption to activity implementation.
	10. I am capable of effectively reducing inappropriate behavior among children in the classroom or using subtle cues to correct such behavior.
	11. I can arrange learning or activity schedules based on children’s interests.
	12. I can focus on learning objectives, using strategies such as summarizing and reorienting goals to guide children’s learning.
	13. I can establish effective classroom management strategies to maximize children’s learning time.
	14. I can ensure children adhere to classroom rules and effectively complete classroom management tasks.
	15. I can establish appropriate routines to ensure smooth transitions and the orderly conduct of activities
Efficacy for child engagement (CE)	16. I can involve children in discussing and formulating class rules.
	17. I can involve children in decisions related to their daily lives.
	18. I can increase the participation of children who are uninterested in activities or have lower ability levels.
	19. I can allow children to choose play materials, peers, and methods independently.
	20. I can fully consider the learning and development needs of group children and the special needs of individual children.
	21. I can organize group activities for all children while also permitting some children to choose and participate in suitable activities according to their own preferences.

### Appendix A.3

**Table A3:** Original version of the OSTES-24 (not formally validated).

	24 Items
Efficacy for instructional strategies (IS)	1. To what extent can you use a variety of assessment strategies?
	2. To what extent can you provide an alternative explanation or example when students are confused?
	3. To what extent can you craft good questions for your students?
	4. How well can you implement alternative strategies in your classroom?
	5. How well can you respond to difficult questions from your students?
	6. How much can you do to adjust your lessons to the proper level for individual students?
	7. To what extent can you gauge student comprehension of what you have taught?
	8. How well can you provide appropriate challenges for very capable students?
Efficacy for classroom management (CM)	9. How much can you do to control disruptive behavior in the classroom?
	10. How much can you do to get children to follow classroom rules?
	11. How much can you do to calm a student who is disruptive or noisy?
	12. How well can you establish a classroom management system with each group of students?
	13. How well can you keep a few problem students from ruining an entire lesson?
	14. How well can you respond to defiant students?
	15. To what extent can you make your expectation clear about student behavior?
	16. How well can you establish routines to keep activities running smoothly?
Efficacy for student engagement (SE)	17. How much can you do to get students to believe they can do well in schoolwork?
	18. How much can you do to help your students value learning?
	19. How much can you do to motivate students who show low interest in schoolwork?
	20. How much can you assist families in helping their children do well in school?
	21. How much can you do to improve the understanding of a student who is failing?
	22. How much can you do to help your students think critically?
	23. How much can you do to foster student creativity?
	24. How much can you do to get through to the most difficult students?
